# The Yin/Yan of CCL2: a minor role in neutrophil anti-tumor activity in vitro but a major role on the outgrowth of metastatic breast cancer lesions in the lung in vivo

**DOI:** 10.1186/s12885-017-3074-2

**Published:** 2017-01-31

**Authors:** Nicole Lavender, Jinming Yang, Sheau-Chiann Chen, Jiqing Sai, C. Andrew Johnson, Philip Owens, Gregory D. Ayers, Ann Richmond

**Affiliations:** 1 Department of Veterans Affairs, Tennessee Valley Healthcare System, Nashville, TN USA; 2 Department of Cancer Biology, Vanderbilt University Medical Center, 432 Preston Research Building, 2220 Pierce Avenue, Nashville, TN 37232 USA; 3 Department of Biostatistics, Vanderbilt University, Nashville, TN USA; 4 Division of Cancer Biostatistics, Department of Biostatistics, Center for Quantitative Sciences, Nashville, TN USA

**Keywords:** CCL2, Breast cancer, Neutrophil killing, Metastasis

## Abstract

**Background:**

The role of the chemokine CCL2 in breast cancer is controversial. While CCL2 recruits and activates pro-tumor macrophages, it is also reported to enhance neutrophil-mediated anti-tumor activity. Moreover, loss of CCL2 in early development enhances breast cancer progression.

**Methods:**

To clarify these conflicting findings, we examined the ability of CCL2 to alter naïve and tumor entrained neutrophil production of ROS, release of granzyme-B, and killing of tumor cells in multiple mouse models of breast cancer. CCL2 was delivered intranasally in mice to elevate CCL2 levels in the lung and effects on seeding and growth of breast tumor cells were evaluated. The TCGA data base was queried for relationship between CCL2 expression and relapse free survival of breast cancer patients and compared to subsets of breast cancer patients.

**Results:**

Even though each of the tumor cell lines studied produced approximately equal amounts of CCL2, exogenous delivery of CCL2 to co-cultures of breast tumor cells and neutrophils enhanced the ability of tumor-entrained neutrophils (TEN) to kill the less aggressive 67NR variant of 4T1 breast cancer cells. However, exogenous CCL2 did not enhance naïve or TEN neutrophil killing of more aggressive 4T1 or PyMT breast tumor cells. Moreover, this anti-tumor activity was not observed in vivo. Intranasal delivery of CCL2 to BALB/c mice markedly enhanced seeding and outgrowth of 67NR cells in the lung and increased the recruitment of CD4+ T cells and CD8+ central memory T cells into lungs of tumor bearing mice. There was no significant increase in the recruitment of CD19+ B cells, or F4/80+, Ly6G+ and CD11c + myeloid cells. CCL2 had an equal effect on CD206+ and MHCII+ populations of macrophages, thus balancing the pro- and anti-tumor macrophage cell population. Analysis of the relationship between CCL2 levels and relapse free survival in humans revealed that overall survival is not significantly different between high CCL2 expressing and low CCL2 expressing breast cancer patients grouped together. However, examination of the relationship between high CCL2 expressing basal-like, HER2+ and luminal B breast cancer patients revealed that higher CCL2 expressing tumors in these subgroups have a significantly higher probability of surviving longer than those expressing low CCL2.

**Conclusions:**

While our in vitro data support a potential anti-tumor role for CCL2 in TEN neutrophil- mediated tumor killing in poorly aggressive tumors, intranasal delivery of CCL2 increased CD4+ T cell recruitment to the pre-metastatic niche of the lung and this correlated with enhanced seeding and growth of tumor cells. These data indicate that effects of CCL2/CCR2 antagonists on the intratumoral leukocyte content should be monitored in ongoing clinical trials using these agents.

**Electronic supplementary material:**

The online version of this article (doi:10.1186/s12885-017-3074-2) contains supplementary material, which is available to authorized users.

## Background

C-C chemokine ligand 2 (CCL2), also known as MCP-1, was first described as a gene induced in response to platelet-derived growth factor that encodes monocyte chemoattractant protein-1 [1, 2]. This chemokine mediates its actions by binding to C-C chemokine receptor 2 (CCR2), a seven-transmembrane G-protein coupled receptor [3]. Though CCL2 affects multiple cell types, its affects mediated through neutrophils or macrophages can be quite different in the presence or absence of activation of TGFβ signaling [4]. CCL2 is both positively and negatively associated with the growth of several tumor types, including breast cancer [5, 6].

The effect of CCL2 on tumor growth and metastasis has been linked to its role in the recruitment of pro-tumor or anti-tumor leukocytes into the tumor microenvironment. CCL2 has been reported to recruit myeloid-derived suppressor cells and pro-tumorigenic macrophages into the tumor microenvironment [6, 7], to promote the invasive and metastatic properties of solid tumors. CCL2 secreted by endothelial cells has been found to stimulate angiogenesis, and ultimately support tumor progression [8]. A recent report by Kitamura et al. also found that CCL2 stimulates breast cancer metastasis through the recruitment of macrophages via CCR2 signaling, followed by a CCL3 mediated enhancement of invasion [9]. Estrogen receptor (ER) negative breast cancers exhibit increased expression of inflammatory chemokines CCL2, CCL4, and CXCL8 compared to ER+ breast cancers and this correlates with the phenotype of the inflammatory infiltrate in the tumor [10]. In an immunohistochemical analysis of CCL2 expression in 205 breast cancer patients, CCL2 was lower in those tumors with ER and progesterone receptor (PR) positivity and higher in basal like breast cancer [11].

While some reports imply that CCL2 can slow tumor progression and metastasis, data from multiple laboratories indicate that inhibiting CCL2 will alter the tumor microenvironment and antagonize tumor growth. The capacity of CCL2 to attract tumor-promoting and immunosuppressive cells or their precursors provides a strong rationale for attempting to therapeutically reduce CCL2 levels in the setting of established neoplasms [12]. Indeed, CCL2 and CCR2 antagonists are currently in clinical trials for treatment of solid tumors in combination with standard chemotherapy (NCT01204996) and for metastatic cancers (NCT01015560, NCT02723006) [13].

Depending on whether CCL2 recruits pro-tumor or anti-tumor neutrophils and monocytes to the tumor will positively or negatively effect tumor growth [14, 15]. CCL2 may attract anti-tumor immune cells that are required for efficient immunosurveillance, such that inhibition of CCL2 may promote neo-carcinogenesis as well as the development of metastases. MMTV-PyMT mice with a genetic deletion of either CCL2 or CCR2 exhibited earlier onset of tumor growth and increased metastasis, though the rate of primary tumor growth was enhanced, implying an anti-tumor role for CCL2 in early stages of tumor progression and in metastasis [16]. Moreover, CCL2 was been reported to increase the cytotoxicity of neutrophils against murine and human breast cancer models, an activity referred to as ‘entrainment’ [17]. When CCL2 was added to co-cultures of naive neutrophils isolated from non-tumor bearing BALB/c mice and 4 T1 cells, tumor cell killing by neutrophils was increased. This same effect was observed when neutrophils were isolated from healthy volunteers and cultured with MDA-MB-231 cells and CCL2 [17]. The same report also demonstrated that neutrophils isolated from tumor bearing mice and patients possess higher levels of CCL2, which contributed to their killing ability. Tumor “entrained” neutrophils (TEN) were reported to kill tumor cells through direct contact in an NADPH Oxidase-H_2_O_2_-dependent mechanism [17]. Thus it is possible that CCL2 can enhance neutrophil-mediated killing of tumor cells.

Based on these conflicting data, we wanted to further evaluate whether CCL2 can “entrain” naïve neutrophils to enhance tumor cell killing using three different tumor models (i.e., 4T1, 67NR, and PyMT). These models were chosen for their varied aggressiveness, comparing the metastatic 4T1 and PyMT cell line with the non-metastatic 67NR cell line. We observed in vitro that CCL2 did increase killing by TEN but not naïve neutrophils in less aggressive 67NR models. However, CCL2 did not enhance killing of 4T1 or PyMT tumor cells by naïve or TEN. Although naïve neutrophils isolated from one mouse genetic background did kill tumor cells derived from another genetic background, exogenous addition of CCL2 did not affect this cytotoxicity. Importantly, intranasal delivery of CCL2 increased the recruitment of leukocytes into the BAL fluid and increased subsets of T cells in the lung, but enhanced the outgrowth of the 67NR breast cancer cells in the lung. Taken together, our findings suggest that CCL2 may have a more pro-tumor effect on tumor growth than an anti-tumor effect.

## Methods

### Cell lines and animals

4T1 (ATTCC-CRL-2539) were obtained from ATCC and the 67NR cells were obtained through an materials transfer agreement from the Karmanos Cancer Institute and cultured according to manufacturer’s specifications. MMTV-PyMT cells were derived from FVB or C57BL/6 mouse strains and passaged in DMEM supplemented with 5% FBS. The more metastatic TGFβR2KO PyMT cells (TbR2KO), isolated from both FVB and C57BL/6 mice were developed in the laboratory of Hal Moses (Vanderbilt University) [18, 19]. The less aggressive TGFβR2^WT^ PyMT and the more aggressive TGFβR2KO PyMT cells were evaluated on mouse backgrounds that are permissive (FVB) and less permissive (C57BL/6) to tumor growth [20]. To selectively determine tumor cell killing, tumor cells were transfected with a GFP2-Firefly luciferase vector. BALB/c, FVB, and C57BL/6 mice were purchased from Charles River Laboratories (Charleston, SC). All animal experiments were approved by the ethics committee of the Vanderbilt Institutional Animal Care and Use Committee review board and were conducted under protocol M/13/052 in compliance with guidelines set forth by the US Department of Health and Human Services Guide for the Care and use of Laboratory Animals.

### Neutrophil isolation

Neutrophils (naïve or TEN) were isolated from the peritoneal wash of BALB/c, FVB, or C57BL/6 mice aged 6–8 weeks using Histopaque-1077 and −1119 (Sigma-Aldrich, Saint Louis, MO). The peritoneal wash was layered on top of Histopaque mediums and spun at 700 g for 30 min without brake. The PMN layer was collected at the interface of Histopaque-1077 and −1119, washed with PBS and re-suspended in Opti-MEM with 0.5% FBS. The isolated cells were >95% neutrophils. Cultures of tumor cells alone, naïve or TEN neutrophils alone, and tumor cells + naïve or TEN neutrophils were seeded into 12-well plates and incubated overnight at 37 °C. A dose response curve was performed to determine the optimal ratio of neutrophils to tumor cells for killing. The maximal ratio for detection of tumor cell killing occurred with a ratio of 30 neutrophils to 1 luciferase expressing tumor cell (30:1). Co-cultures of neutrophils and tumor cells were incubated for 18 h in the presence and absence of 50 ng/mL CCL2 (R&D Systems, Minneapolis, MN) or 50 ng/mL CCL2-neutralizing antibody (BD Biosciences, #554440 San Jose, CA).

### FACS analysis of neutrophil content and CCR2 expression

To prepare single cell suspensions tumors were diced, processed using gentle MACS dissociator (Miltenyi Biotec) and subjected to enzymatic digestion with 1500 CDU Collagenase I, 1 mg/mL Dispase II, and 0.01 MU DNase I per sample for 1 h. Cell suspensions were strained through 70 μm nylon mesh. Samples were washed with PEB buffer (0.5% BSA in PBS) and 1x10^6^ cells from each sample were stained with antibody cocktail (CD45-APC/Cy7 (Biolegend, # 103116, San Diego, CA), CD11b-FITC (BD Pharmingen, #553310, San Jose, CA), Ly6G-PE (BD Pharmingen, #551461, San Jose, CA). The amount of each antibody to use was determined based on prior titration experiments. Purified anti-mouse CD16/CD32 antibody (BD Pharmingen, #553142 San Jose, CA) was added to prevent non-specific antibody binding. After 30 min incubation with antibodies, cells were washed twice with PEB buffer, fixed in 0.5% buffered PFA and analyzed on a custom 5-laser LSRII (BD Biosciences, San Jose, CA).

### ELISA assays

After incubation of neutrophils alone or tumor cells alone for 18 h, media were collected from cell cultures and stored at 4 °C until subjected to ELISA assay for murine CCL2. All ELISAs were preformed according to the manufacturer’s instructions (R&D Systems, Minneapolis, MN).

### Luciferase reporter killing assays

For reporter assays, luciferase expressing tumor cells were washed with 1X PBS buffer after removing media, then lysed using Promega Reporter Lysis Buffer (Luciferase Assay System, Promega, Madison, WI). Cell lysates were transferred from plates to microcentrifuge tubes, and spun to remove remaining cellular debris. Subsequently, 20 μl of cell lysate supernates were pipetted into opaque 96-well plates, mixed with Luciferase Substrate (20 μl of Luciferin), and luminescence was read immediately for 10 s with a Luminescence reader (Promega, Madison, WI).

### Determination of Reactive Oxygen Species (ROS) and Granzyme-B Release

ROS was measured by L-012 (Wako Chemicals USA, Inc, Richmond, VA) or Luminol (Fisher Scientific, Sewanee, GA). For L-012 assays, media from single and co-cultured samples was collected after the 18 h incubation period. Samples were seeded into an opaque 96-well plate with L-012 in the absence or the presence of Catalase. Luminol experiments were performed with isolated neutrophils (naïve or TEN) that were immediately seeded into opaque plates and incubated with Luminol at room temperature for 15 min. Stimulants were then added and luminescence was measured over a 10 min period. For both assays, samples were protected from light and read on luminometer. Granzyme-B release was measured by ELISA (R&D Systems, Minneapolis, MN) using conditioned media collected after overnight incubation at 37 °C.

### Intranasal Delivery of CCL2

Mice were anesthetized using an isoflurane vaporizer and then 100 ng of CCL2 in 10 μl of PBS was delivered by the intranasal route. The solution of CCL2 was gently placed on the nares of the mice where it is readily taken in.

### Analysis of outgrowth of 67NR cells in the lung after intranasal delivery of CCL2

1 × 10^6^ 67NR cells were intravenously injected into mice. These mice received intranasal delivery of 100 ng of murine CCL2 daily. After two weeks of CCL2 treatment, mice were sacrificed and lungs were removed, photographed, and weighed. The lung tumor weights were normalized to the weight of tumor-free lungs.

### Analysis of BAL Fluid Leukocytes after Intranasal Delivery of CCL2

Murine leukocytes were isolated and subsets analyzed by FACS as we have previously described [21, 22] (see reference 18 Supplemental Data for a complete listing of antibody sources). CCR2 expression in BALB/c and FVB neutrophils was determined by FACS analysis using PE-conjugated anti-CCR2 from R&D Systems, Minneapolis, MN.

### Analysis of the ability of less aggressive PyMT breast tumors in the mammary Fat Pad to reduce the lung colonization of more aggressive TGFβR2 knock Out PyMT tumors after tail vein injection

Female FVB mice (10 weeks old) were injected into the 4^th^ mammary fat pad (MFP) with either PBS alone or PBS containing 15,000 PyMT breast cancer cells. Two weeks later when the tumor was palpable, either PBS alone (MFP-PBS) or 1 × 10^6^ TGFβR2 knockout PyMT breast cancer cells in 200 μl of PBS (MFP + TbR2KO) were delivered to the tumor-bearing mice by tail vein injection. A third group of mice (non-tumor bearing) received 1 × 10^6^ TGFβR2KO PyMT cells via tail vein (t.v.) injection (t.v. TbR2KO). Three weeks later, mice were sacrificed and lungs were removed, weighed, fixed in paraformaldehyde, embedded in paraffin, subjected to H&E staining, then the number of metastases counted.

### Statistical analyses

**Fig. 1:**
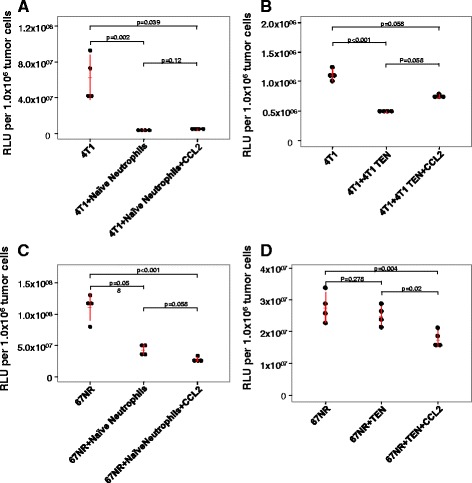
CCL2 enhances killing of 67NR cells but not 4 T1 cells by neutrophils. Tumor cells were seeded with and without neutrophils at a ratio of 30 neutrophils to 1 tumor cell in the absence and presence of CCL2. After 18-h incubation at 37 °C, cells were lysed and luciferase was measured to determine tumor cell killing. Luminescence was analyzed using the Kruskal-Wallis (KW) test with Dunn’s post-test if the KW test was statistically significant (*p* < 0.05). **a** & **b** Naïve as well as tumor entrained neutrophils were able to kill 4 T1 tumor cells (*p* = 0.002 and *p* < 0.001, respectively). **c** Naïve neutrophil killing of 67NR cells resulted in a *p* value of 0.058, but the addition of CCL2 resulted in a statistically significant killing of 67NR cells (*p* = 0.001) **d**. TEN were not capable of killing tumor cells, but addition CCL2 to TEN enhanced this effect in 67NR models (p-0.02 for 67NR + TEN vs. 67NR + TEN + CCL2, *p* = 0.004 for 67NR vs. 67NR + TEN + CCL2) Kruskal-Wallis test with Dunn’s test for multiple comparisons. Values are graphed as mean ± SD

**Fig. 2:**
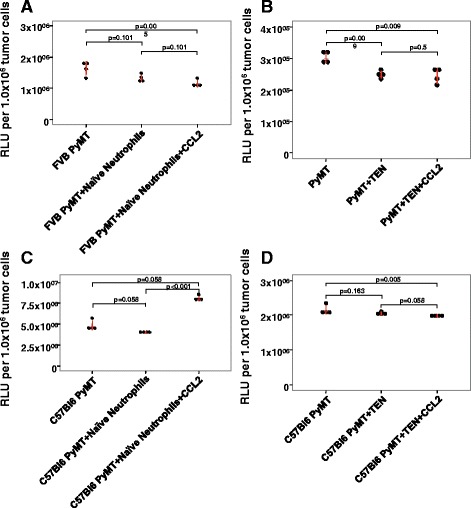
Naïve or tumor entrained neutrophils (TENs) are able to kill PyMT tumor cells in vitro, but CCL2 does not increase this effect. PyMT cultures were seeded the same as 4T1 and 67NR with the exception that neutrophils were isolated from either FVB or C57BL/6 mice, depending on cell line background. **a** Naïve neutrophils from FVB mice were not capable of killing tumor cells, but CCL2 addition to these naïve neutrophils significantly killed tumor cells (*p* = 0.005). **b** TENs were capable of killing tumor cells (*p* = 0.009). However, CCL2 did not significantly increase killing (*p* = 0.5). **c** Naïve neutrophils from C57BL/6 mice did not kill C57BL/6 PyMT cells (*p* = 0.058), and CCL2 addition to this co-culture enhanced the number of viable PyMT tumor cells (*p* < 0.001). **d** C57BL/6 TEN had little effect on the viability of autologous PyMT tumor cells, but CCL2 addition to these TENs resulted in a modest reduction in viable PyMT tumor cells (*p* = 0.005). Kruskal-Wallis test with Dunn’s test for multiple comparisons. Values are graphed as mean ± SD

**Fig. 3:**
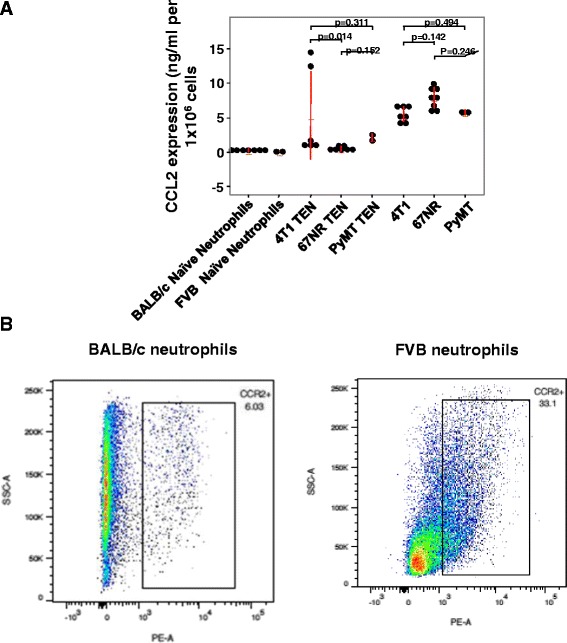
Tumor cells secrete significantly higher levels of CCL2 compared to naïve and tumor entrained neutrophils. **a** Conditioned media was collected from cultured cells after 18-h incubation at 37 °C, and then CCL2 levels were analyzed by ELISA. Tumor cells tended to secrete higher levels of CCL2 than naïve neutrophils, but there were no statistical differences among the groups. Kruskal-Wallis test with Dunn’s test for multiple comparisons; mean ± SD are graphed. **b** CCR2 expression on neutrophils isolated from BALB/c versus FVB mice. Membrane expression of CCR2 was evaluated on neutrophils isolated from BALB/c and FVB mice using protocols described in Methods using PE-conjugated anti-murine CCR2. While CCR2 was expressed by only 6.03% of the neutrophils from BALB/c mice, 33.1% of the neutrophils from FVB mice expressed cell surface CCR2

**Fig. 4:**
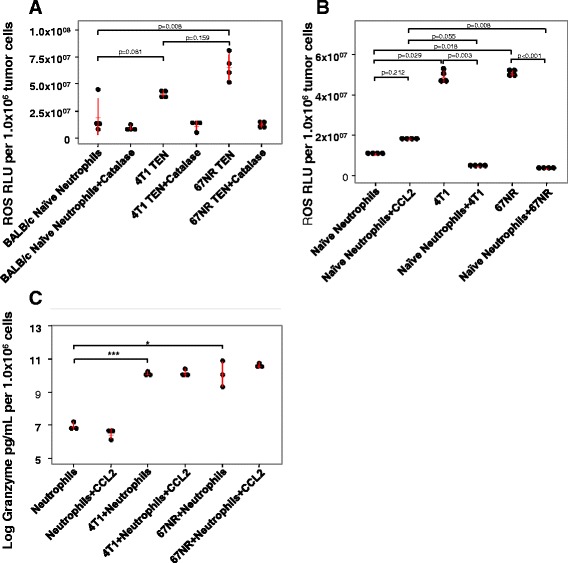
Tumor entrained neutrophils produce greater amounts of ROS than naïve neutrophils. **a** Conditioned media was collected after overnight incubation and tested for ROS using L-012 luminescent probe. Since this reagent measures all ROS, the addition of catalase determined the presence of hydrogen peroxide. 67NR TEN produce more ROS, including hydrogen peroxide, than naïve neutrophils (*p* = 0.008). **b** ROS Levels do not correlate with tumor cell killing. Intra- and extracellular ROS were measured in the Luminol assay. Single cell suspensions of tumor cells or freshly isolated naïve neutrophils were incubated with Luminol for 15 min at room temperature. CCL2 or tumor cells were then added to naïve neutrophils and luminescence was immediately measured. CCL2 did not significantly increase the ROS signal (*p* = 0.212); moreover, a decrease in ROS signal was observed when tumor cells and neutrophils were co-cultured (*p* = 0.003 for 4 T1 vs. Neutrophils + 4 T1 and *p* < 0.001 for 67NR vs. Neutrophils + 67NR). **c** Granzyme-B release when neutrophils are co-cultured with tumor cells and CCL2. Granzyme-B levels in conditioned media from cultured cells were determined by ELISA. 4T1 cells co-cultured with naïve neutrophils exhibited higher granzyme-B release than neutrophils alone (adj. *p* = 0.025). Also 67NR cells co-cultured with naïve neutrophils resulted in a significant increase in granzyme-B release over that of neutrophils alone (adj. *p* < 0.001). For Fig. 4a and b, Kruskal-Wallis test with Dunn’s test for multiple comparisons. For Fig. 4c, a log transformation was used to meet the normality assumption. ANOVA for an overall comparison and *t*-test for multiple comparisons with Bonferroni *p*-value adjustment was used. Values are graphed as mean ± SD

**Fig. 5:**
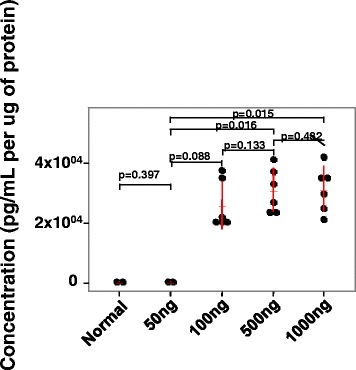
Intranasal delivery of CCL2 increases the concentration of CCL2 in the lung. Daily intranasal delivery of increasing concentrations of CCL2 (0, 50, 100, 500 and 1000 ng) resulted in increasing concentrations of CCL2 in the lung with saturation achieved by the 500 ng delivery or 1000 ng delivery compared to 50 ng delivery (*p* = 0.016 and *p* = 0.015, respectively). Kruskal-Wallis test with Dunn’s test for multiple comparisons. Data are graphed as mean ± SD

**Fig. 6:**
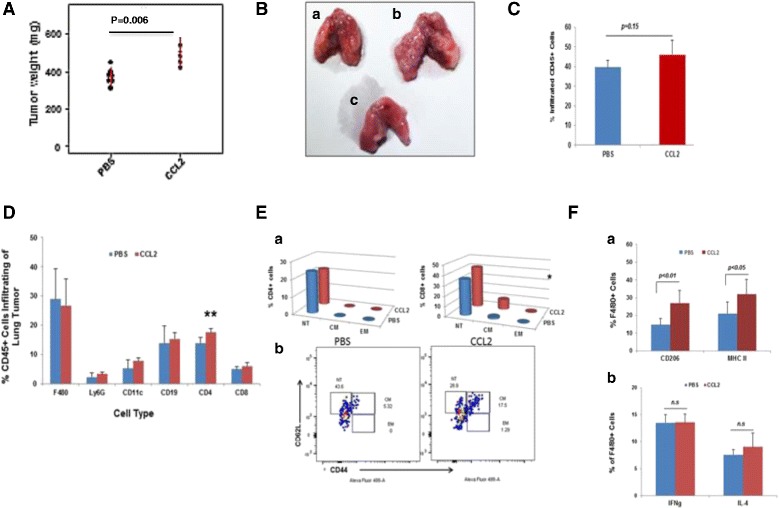
CCL2 promotes lung tumor growth. **A** BALB/c mice were intravenously implanted with 1 × 10^6^ 67NR cells and 100 ng CCL2 cytokine or PBS vehicle were delivered daily by the intranasal route for 5 days/week for 2 weeks. Two weeks after treatment, mice were sacrificed and the lung weight was determined. Wilcoxon rank sum test, *p* = 0.006. Mean ± SD. **B** Photographs of Lungs from CCL2 or PBS treated mice prior to *i.v.* delivery of 1 × 10^6^ 67NR cells. Lungs from mice in Fig. 6a were removed from euthanized mice and representative ones were photographed. PBS-treated mouse lung (a), CCL2-treated mouse lung (b), or tumor-free un-treated lung (c). **C** Lungs from CCL2-treated mice do not exhibit significant increase infiltrate of CD45+ cells. Mice treated as described in 6A were euthanized; lungs were harvested then prepared for FACS analysis of infiltrating CD45+ leukocytes. Data are reported as % of CD45+ cells total lung cells analyzed. Student’s *t*-test, *p* = 0.15. **D** CCL2 enhanced immune cells infiltrating into lung tumor. BALB/c mice were treated as described in 6a. Two weeks after treatment, the lung tumor microenvironment was analyzed for the infiltration of immune cells by multicolor FACS. Data were analyzed by Student’s *t*-test. **p < 0.01, n = 5. **E** CCL2 increases central memory CD8+ T cells but not effector memory T cells. **Ea** BALB/c mice were treated as described in 6A. Two weeks after treatment, the lung tumor microenvironment was analyzed for memory T cells by multicolor FACS. Data were analyzed by the Student’s *t*-test. *p < 0.05 vs. PBS controls, *n* = 5. **Eb** a representative graph indicating CD44 and CD62L expression on CD8+ T cells from PBS-treated mouse lung or from CCL2-treated murine lung. **F** CCL2 effects on the polarization of lung macrophages. BALB/c mice were treated as described in 6A. Two weeks after treatment, the lung tumor microenvironment was analyzed by FACS for **a**) the infiltration of F4/80+ macrophages expressing CD206 (**p < 0.01, *n* = 5.) and MHC II markers on the cell surface (* *p* < 0.05, *n* = 5) (or **b**) for the intracellular cytokines IFNγ and IL-4 (ns = not significantly different)). Data were analyzed by the Student’s *t*-test, *n* = 5

The Kruskal-Wallis (KW) test, a nonparametric analog of analysis of variance, was performed to test for an overall difference among groups for Luciferase Reporter Assays and ELISAs (Figs. 1, 2, 3, and 4a, b, and 5). Dunn’s post-test was used for pair-wise multiple comparison among groups if the KW test was statistically significant (*p* < 0.05). Analysis of variance with a Bonferroni correction for multiple comparisons was used in Fig. 4c due to a decrease in sample size. The Wilcoxon rank sums test was used to test for statistically significant differences in tumor weight between PBS and CCL2 treated tumor-bearing mice (Fig. 6). Analysis of variance with blocking (two experiments) was performed to test for an overall difference in number of lung metastasis among MFP-PBS, MFP + TbR2KO tail vein injected (t.v.), and TbR2KO groups, t.v. injected alone groups. Tukey’s honestly significant difference (HSD) was used for pair-wise multiple comparisons. The log rank test was performed to test for differences in the distributions of relapse-free-survival (RFS) and CCL2 expression (i.e., high versus low) among all breast cancers as well as within several the subtypes of breast cancer, respectively. Hereafter, * = *p* < 0.05, ** = *p* < 0.01, and *** = *p* < 0.001, respectively.

## Results

### Effects of CCL2 on In vitro killing of tumor cells by naïve neutrophils

To evaluate the capacity of CCL2 to entrain neutrophils to enhance tumor cell killing, we utilized a combination of in vitro experiments with exogenous delivery of CCL2 to co-cultures of neutrophils and either aggressive 4T1 breast cancer cells compared to a less aggressive 4T1 variant, 67NR, or co-cultures of neutrophils with either C576Bl/6 or FVB-PyMT breast tumor cells. This experimental design allowed us to examine the ability of exogenous CCL2 to enhance the ability of naïve neutrophils or TEN to kill luciferase expressing aggressive and less aggressive breast tumor cells. Naïve neutrophils were isolated from non-tumor bearing BALB/c mice (for luciferase expressing 4T1 and 67NR cultures), FVB, or C57BL/6 mice (for PyMT cultures). Both FVB and C57BL/6 mice were used for the PyMT model since the FVB strain is known to be more permissive for tumor growth and C57BL/6 is much less permissive [20, 23–25]. We first determined that the optional ratio of neutrophils to tumor cells was 30:1. When naïve neutrophils from BALB/c mice were co-cultured at a ratio of 30 to 1 with 4 T1 cells, the neutrophils were indeed able to kill the tumor cells based upon a reduction in intracellular luminescence (RLU) comparing tumor cells alone to tumor cells plus neutrophils as illustrated in Fig. 1a (*p* = 0.002). Moreover, addition of CCL2 (50 ng/ml) to co-cultures of naïve neutrophils and 4 T1 cells did not increase the tumor cell killing over that produced by naïve neutrophils without CCL2 addition (Dunn’s test, *p* = 0.12) (Fig. 1a). That is, there was no statistically significant change in luminescent signal between the tumor cells plus naïve neutrophils samples and tumor cells plus naïve neutrophils plus CCL2 samples. Naïve neutrophils from BALB/c mice did not significantly reduce the viability of the 67NR cells based upon RLU measurements (adj. *p* = 0.058) (Fig. 1c), and addition of CCL2 did not significantly change the viability of 67NR cells co-incubated with naïve neutrophils alone (*p* = 0.058) (Fig. 1c). However, co-cultures of 67NR cells and naïve neutrophils treated with CCL2 (50 ng/ml) did significantly reduce the viability of 67NR cells (*p* = 0.001) (Fig. 1c). While naïve neutrophils from FVB mice did not significantly reduce the viability of PyMT tumor cells (*p* = 0.101) (Fig. 2a), addition of exogenous CCL2 did lead to a decrease in viability of the PyMT cells in this co-culture compared to PyMT cells without neutrophils (*p* = 0.005) (Fig. 2a). In the C57BL/6 PyMT model, there was no reduction in viability of the PyMT cells upon incubation with naïve neutrophils from C57BL/6 (adj. *p* = 0.058) and we observed enhanced tumor cell viability in co-cultures of naïve neutrophils treated with CCL2 (adj. *p* = 0.001) (Fig. 2c).

### Effects of CCL2 on In vitro tumor cell killing by tumor entrained neutrophils

We next performed these experiments using neutrophils isolated from mice bearing 4T1, 67NR, or PyMT tumors, which we refer to as tumor entrained neutrophils (TEN). TENs from BALB/c or FVB mice were able to kill 4T1 tumor cells and PyMT tumor cells, respectively in vitro (*p* < 0.001 and *p* = 0.009, respectively) (Figs. 1b and 2b). When we cultured 4T1 tumor cells with TEN, we observed a >56% reduction in luminescent signal, indicating that as with naïve neutrophils from BALB/c mice, TENs were also able to kill 4T1 cells (adj. *p* = 0.058) (Fig. 1b). As we saw with naïve neutrophils, exogenous CCL2 did not enhance tumor cell killing by BALB/c TEN (adj. *p* = 0.058) (Fig. 1b). In the case of 67NR cells, TENs were not effective at killing tumor cells (*p* = 0.278) (Fig. 1d). However, the addition of CCL2 did increase tumor cell killing by the TENs in these co-cultures over that by the TENs alone (*p* = 0.005) (Fig. 1d). In PyMT mouse models, TENs from FVB mice were able to kill PyMT tumor cells (adj. *p* = 0.028), but exogenous CCL2 did not increase that killing (*p* = 0.50) (Fig. 2b). In contrast, with the C57BL/6 PyMT model, TEN did not significantly reduce PyMT tumor cell viability, but addition of CCL2 to these co-cultures resulted in a very small but significant change in tumor viability based upon cell luminescence compared to the PyMT cells not cultured with TENs (*p* =0.005) (Fig. 2d). However, CCL2 did not enhance killing of C57BL/6 TEN neutrophils co-cultured with PyMT tumor cells compared to PyMT plus TEN alone (Fig. 2d).

We did not observe any biologically significant increase in tumor cell killing in response to CCL2 with 4T1 tumor cells, likely because the naïve neutrophils and TEN alone killed most of the 4T1 tumor cells, leaving little room for enhanced killing. Moreover, the increases in TEN and naïve neutrophil killing in response to CCL2 for PyMT cells in FVB or C57BL/6 models were minimal. One possibility considered to explain these differences in tumor cell killing ability was that naïve neutrophils isolated from BALB/c mice are more effective than FVB or C57BL/6 neutrophils in vitro, particularly in less aggressive models. To determine whether the naïve neutrophils from BALB/c are more aggressive in killing than those of C57BL/6 mice, we tested the ability of naïve BALB/c neutrophils to kill PyMT tumor cells from the FVB mouse background (Additional file 1: Figure S1). We found that naïve neutrophils isolated from BALB/c mice are indeed able to kill PyMT tumor cells in vitro (*p* = 0.005), but exogenous CCL2 dids not enhance killing (*p* = 0.347). This implies that there may be something different about naïve BALB/c neutrophils as compared to FVB or C57BL/6 naïve neutrophils with regard to their ability to kill tumor cells. However, the ability of CCL2 to increase TEN killing appears to be limited to less aggressive 67NR cells.

### Assays to evaluate factors in conditioned media that affect neutrophil anti-tumor activity

Since the effects of CCL2 on TEN appeared to be limited to less aggressive tumor cells, we examined whether differences in CCL2 secretion may influence the response to exogenous CCL2 to enhance tumor cell killing. Neutrophils isolated from naïve or tumor bearing BALB/c or FVB, as well as tumor cells, were seeded into 6-well plates and incubated for 18 h. Conditioned media were collected and the CCL2 level was measured by ELISA. In these experiments, naïve and TEN neutrophils produced very low levels of murine CCL2 (with the exception of 2/5 isolates of 4 T1 TEN), while tumor cells tended to secrete much higher levels of CCL2. However, there were no statistical differences in secretion of CCL2 between 4T1, 67NR or PyMT cells or between BALB/C and FVB neutrophils (Fig. 3). Interestingly, co-culture of naïve or TEN with tumor cells resulted in a decrease in CCL2 in the media as detected by ELISA, but there was an increase in the amount of CCL2 in the cell lysate (data not shown). This disparity is likely because the CCL2 produced by the tumor cells was taken up by the neutrophils. Moreover, there may have been an increased production of CCL2 by the cells in co-culture that was not secreted. Thus, differences in ability to respond to exogenous CCL2 did not result from differences in the levels of CCL2 produced by tumor cells or neutrophils (naïve or TEN) isolated from BALB/c or FVB mice. However, addition of anti-CCL2, but not isotype matched control IgG, was able to reverse the naïve neutrophil killing of 67NR cells, indicating that CCL2 was needed for the neutrophil killing of tumor cells (Additional file 1: Figure S4). We also examined the level of CCR2 expression on naïve neutrophils isolated from BALB/c mice as compared to naïve neutrophils from FVB mice. We observed CCR2 receptor levels were higher in the FVB neutrophils, indicating that the failure of the FVB neutrophils to respond to exogenous CCL2 with enhanced tumor cell killing was not due to a lack of CCR2 receptor expression (Fig. 3b).

### Evaluation of CCL2 effects on neutrophil ROS and granzyme-B release

Neutrophils are able to kill tumor cells and invading pathogens by several means. This includes production of reactive oxygen species (ROS) and release of lytic enzymes from granules [26]. To examine the killing mechanisms in our cell cultures, we tested for ROS production using L-012 and/or Luminol as well as granzyme-B secretion via ELISA. We found that 67NR TENs but not 4T1 TENs produce more ROS than naïve BALB/c neutrophils, as shown in Fig. 4a (*p* = 0.008 and *p* = −.081, respectively). The addition of catalase to these conditioned media samples caused a decrease in ROS, illustrating naïve neutrophils and TENs are producing hydrogen peroxide. Despite the higher levels of ROS produced, this did not correlate with increased tumor cell killing in luciferase reporter assays (Fig. 1). That is, 4T1 TENs and 67NR TENs were less efficient at killing tumor cells than naïve neutrophils (Fig. 1). 4T1 TENs were able to reduce tumor cell viability by roughly 50% (*p* < 0.001, Fig. 1b), while naïve BALB/c neutrophils were able to kill nearly 100% of the tumor cells (*p* = 0.002, Fig. 1a). We then examined intracellular and extracellular ROS in naïve neutrophils (Fig. 4a and b). This analysis revealed that 67NR TENs possess significantly higher levels of ROS than naïve neutrophils (*p* = 0.008 for 67NR TEN vs. BALB/c neutrophils) (Fig. 4a). Moreover, tumor cells produced more ROS than naïve neutrophils (*p* = 0029 for 4T1 vs. neutrophil, *p* = 0.018 for 67NR vs. neutrophil) (Fig. 4b), and addition of naïve neutrophils to 4T1 cells or 67NR cells actually decreased ROS (*p* = 0.003 and *p* < 0.001, respectively) (Fig. 4b), likely due to loss of tumor cell ROS due to tumor cell killing. Also, addition of naive neutrophils to 67NR cells resulted in ROS levels that were much lower than those produced when CCL2 was added to naïve neutrophils (*p* = 0.008) (Fig. 4b). Hence, ROS detection as measured here correlates with tumor cell killing only in the sense that when naïve neutrophils kill 4T1 or 67NR cells, there is a concordant reduction of ROS, since the ROS is mainly derived from the tumor cells. We postulated the killing mechanism utilized by naïve and TEN likely involves mechanisms other than induction of ROS. Consequently, we examined granzyme-B release in conditioned media collected from cell cultures (Fig. 4c). When 4T1 tumor cells were added to naïve neutrophils we observed increased granzyme-B release compared to neutrophils alone (adj. *p* = 0.025, Fig. 4c). Also 67NR cells co-cultured with naïve neutrophils resulted in a significant increase in granzyme-B release over that of naïve neutrophils alone (adj. *p* < 0.001) (Fig. 4c). These data indicate that neutrophil killing of 4T1 and 67NR cells was associated with granzyme-B activity. However, addition of exogenous CCL2 did not increase that granzyme-B activity.

### Effect on less aggressive breast cancer implants on the colonization of more aggressive breast cancer cells

Granot et al. argued that CCL2 produced by tumor cells could both enhance the growth of the primary tumor and at the same time entrain neutrophils in the lung to kill tumor cells and inhibit lung metastasis [17]. Since we observed that the PyMT tumor cells make a substantial amount of CCL2 (Fig. 3), as Granot argued, it could be postulated that CCL2 released into the blood stream by a primary PyMT tumor might impair the outgrowth of tumor cells in the lung after intravenous injection. TGFβ is known to suppress CCL2 expression, thus it is expected that TGFβR2KO PyMT cells will express more CCL2 and thus provide a good model for exploring the role of CCL2 production by tumor cells in metastasis. Fridlender et al. showed that TGFβ has the ability to inhibit the anti-tumor activity of TENs [4], thus we reasoned that loss of response to TGFβ by PYMT cells should allow for increased CCL2 production and enhanced anti-tumor activity of TEN at the pre-metastatic site. We tested this hypothesis by implanting 15,000 PYMT tumor cells into the 4^th^ mammary fat pad (MFP) and when palpable tumors developed in the MFP, the tumor bearing mice received intravenous injection of 1 × 10^6^ of the more aggressive TGFβR2KO PyMT breast tumor cells [27], or vehicle control. A second group of mice did not have tumors implanted into the MFP, but received only the intravenous injection of the TGFβR2KO PyMT tumor cells at the same time as the MFP tumor bearing mice. After allowing two additional weeks for the outgrowth of the intravenously injected tumor cells, all mice were euthanized and the lungs were examined for metastasis based upon visual examination, weight, and histology. Mice that only received an orthotopic implantation of PYMT tumor cells (expressing TGFβR2) in the MFP did not develop tumors in the lung during this period of time. Interestingly, there was a trend toward fewer tumors in the lungs of mice with PyMT tumors growing in the MFP that also received tail vein injections of the TGFβR2KO PyMT tumor cells, as compared to mice that only received the tail vein injection of TGFβR2KO PyMT breast tumor cells (adj. *p* = 0.091) (Additional file 1: Figure S2). These data imply that signals emanating from the orthotopic tumor might indeed have an adverse impact on the colonization of circulating tumor cells. This concept is compatible with the idea that less aggressive tumors may be able to “entrain” the microenvironment in the lung to inhibit the growth of more aggressive tumors. Moreover, we know these tumor cells produce significant amounts of CCL2 (Fig. 3), even though they continue to express TGFβR2. We did not measure the CCL2 levels in the serum or lung after implantation of the TβR2WT PyMT into the MFP as compared to normal lung or lung after tail vein injection of PyMT-TβR2KO alone, so we cannot definitely equate the suppression of tumor outgrowth in the lungs to elevations in CCL2. In fact, other investigators have shown CCL2 elevation in the tumor microenvironment and premetastatic niche enhances tumor growth and metastasis [6, 22].

### In vivo experiments to evaluate How delivery of CCL2 to the lung affects colonization of the lung by breast cancer cells

To evaluate the idea that higher tissue levels of CCL2 might make changes in the microenvironment that can inhibit tumor growth, we delivered increasing amounts of CCL2 intranasally to mice and monitored the concentration of CCL2 in the lung (Fig. 5a). However, the delivery of 100 ng vs 500 or 500 ng vs 1000 ng did not reveal statistically significant differences in the concentration of CCL2 in the lung (*p* = 0.133 and *p* = 0.482, respectively) (Fig. 5a). This may be due to the uptake of exogenous CCL2 by leukocytes and other stromal cells in the lung since endothelial cells express high levels of CCR2 [28]. In contrast, increasing intranasal delivery of CCL2 enhanced recruitment of CD8+ T lymphocytes into the BAL fluid and the number of CD45+ cells in the BAL that were CD8+ tended to show a dose dependent increase. Also there was a tendency to increase Ly6G+/F4/80+ cell recruitment into the BAL fluid in response to increasing delivery of CCL2 (Additional file 1: Figure S3A and B). In the absence of intranasal delivery of CCL2, BAL from PBS controls did not exhibit a measureable lymphocyte population and macrophages constituted <10% of live cells.

To examine the pro-tumor or anti-tumor function of CCL2 in vivo, syngeneic 1 × 10^6^ 67NR cells were intravenously injected into BALB/c mice. Subsequently, 100 ng of CCL2 was delivered daily by the intranasal route for two weeks, then mice were sacrificed and lung tumor burden was examined. After two weeks of exogenous CCL2 delivery, we observed that the net tumor contribution to the weight of the lung in the CCL2 treated group was significantly increased [314 ± 83 mg, *n* = 5] in comparison with the PBS control group [184 ± 45 mg, *n* = 7] (Wilcoxon rank sum test, *p* = 0.006) (Fig. 6a and b, comparing 6Bb to Ba). Thus, exogenous CCL2 favors 67NR tumor colonization in vivo, even though it can enhance 67NR tumor cell killing by TEN in vitro (Fig. 1d). These data point to the significance of the tumor microenvironment with regard to chemokine responses.

When we performed analysis of the leukocyte infiltrate in the lungs of mice given intranasal injection of PBS versus CCL2 (100 ng/ 5 days/week for 2 weeks) prior to receiving tail vein injection of 1 × 10^6^ 67NR cells, we did not observe a significant increase in the CD45 cells recruited to the lung following intranasal delivery of CCL2, though a substantial percentage of the cells in the lung were CD45+ (40-45%) (Fig. 6c, *p* = 0.15). Of the CD45 cells in the lung, ~27% were F4/80+, <5% were Ly6G+, 5-8% were CD11c+, 17-18% were CD19+, 17-18% were CD4+, and ~5% were CD8+ (Fig. 6d). While CCL2 did not significantly affect the total F4/80, Ly6G, CD11c, CD19, and CD8 cell content in the lung as a percentage of the total CD45+ cells, there was a significant increase in the percentage of CD4+ T cells (*p* < 0.01, *n* = 5, Student’s *t*-test) (Fig. 6d). We also observed that CCL2 increased the population of central memory CD8+ T cells (*p* < 0.05, Student’s *t*-test), but did not alter the percentage of CD4+ T cell central memory cells, the effector memory CD4 + T cells, or CD8+ T cells (Fig. 6e). Though CCL2 treatment did not increase the population of F4/80 or Ly6G cells in the tumor, it did increase the percentage of F4/80 cells that expressed CD206, a marker for M2 macrophages. In contrast, CCL2 intranasal delivery increased the F4/80 population expressing MHCII, a marker for M1 macrophages (Student’s *t* test, *p* < 0.01 and *p* < 0.05, respectively) (Fig. 6Fa). There were no significant changes in the population of F4/80 cells producing IFNγ or IL-4, though there was a trend toward increased IL-4 in the CCL2 group (Fig. 6Fb).

### Correlation between CCL2 mRNA expression in sub-classes of human breast cancer and prognosis

**Fig. 7:**
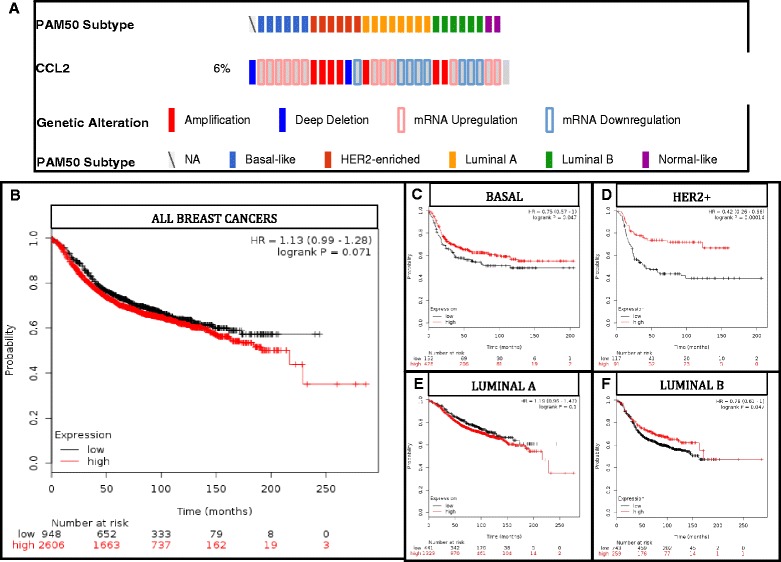
CCL2 Expression in Sub-Groups of Human Breast Cancer Correlates with Relapse-Free Survival. **a** CCL2 expression in the TCGA (The Cancer Genome Atlas) as queried by the cBIO.org portal selected for the Breast Cancer Nature 2012 study containing PAM50 subtyping of breast tumors. **b** Probability of RFS from kmplot.com in comparison to CCL2 expression using median and auto-cutoffs is shown. **c** Basal; **d** HER2+; **e** Luminal A; **f** Luminal B

Another way to examine the impact of CCL2 expression by tumor cells is to determine whether CCL2 expression correlates positively or negatively with relapse free survival. When we examined the TCGA and kmplot.com data base to query expression of CCL2 (i.e., high vs. low expression) in human breast cancer with respect to relapse-free survival (RFS), the association of high CCL2 expression with RFS did not reach statistical significance (*p* = 0.071) (Fig. 7b). However, with some subtypes of breast cancer, patients expressing high levels of CCL2 exhibited improved RFS. For example high CCL2 expression suggested improvement in RFS for basal (*p* = 0.047), HER2+ (*p* < 0.001) and luminal B (*p* = 0.047) breast cancers (Fig. 7c, d and f). However, among patients with luminal A breast cancer, the most abundant of the sub-group, RFS differences among patients with high and low CCL2 expression was equivocal (*p* = 0.1) (Fig. 7e).

## Discussion

CCL2 has been described as both supporting breast cancer growth and progression and inhibiting breast cancer progression. When 4T1E/M3 cells with enhanced capacity to metastasize to bone were altered to increase CCL2 production, there was an inhibition of metastasis of these cells to bone and lung as compared to 4T1E/M3 cells expressing normal amounts of CCL2 [29]. CCL2 may play a protective role by mediating early immune surveillance in the tumor progression process [16] through a process that involves recruitment of γ,δ tumor infiltrating lymphocytes (TILS) to the tumor microenvironment [30]. CCR2, the receptor for CCL2, is expressed on human effector memory CD4+ T cells that are useful for rapid recall responses [31], on Th17 cells recruited to the lung during allergic reactions [32], on γ,δ T-cells infiltrating tumors [30], and on CD4+ T cells where they can have negative influence in Crohn’s disease [33].

Other reports show that CCL2 production by tumor cells impairs T cell-mediated anti-tumor activity [34]. For example, Fujimoto described a role for stromal CCL2 (MCP-1) in the recruitment of tumor promoting macrophages into early breast cancer lesions, a process which promotes tumor progression. MCP-1 mRNA expression was enhanced when stromal cells were co-cultured with breast tumor cells, and when immune deficient mice received blocking antibodies to MCP-1 (CCL2), there was a reduction in angiogenesis, macrophage infiltration into the tumors and also the growth of the tumors slowed. Hembruff et al. showed that TGFβR2 KO in fibroblasts increases CCL2 production and when these fibroblasts are co-implanted with 4T1 cells, there is enhanced primary tumor growth and metastases as compared to 4 T1 cells co-implanted with TGFβR2 expressing fibroblasts [35]. Moreover, the Pollard laboratory has shown that inhibition of CCL2 /CCR2 signaling blocks the recruitment of inflammatory macrophages and reduces metastasis to the lung [6]. Additionally, expression of this chemokine was associated with a poor prognosis in breast cancer [36].

Similar findings were observed with 230 samples of human breast cancer primary lesions where CCL2 expression in tumor cells and accumulating tumor associated macrophages (TAMs), increased angiogenesis, and vessel invasion of tumor cells [37]. In still another study of 427 invasive ductal carcinoma breast cancer cases, the expression of CCL2 in the stroma of basal-like breast cancer correlated with significant reduction in recurrence-free survival [38]. While other studies have demonstrated CCL2 as a prognostic factor by evaluating selected cell populations or distinct location of metastases, we have chosen to look at a large dataset of more than 3000 primary breast cancers to evaluate the overall expression of CCL2 mRNA. While we find that CCL2 cannot be used as a prognostic factor of all breast cancer, but that it can be prognostic for distinct subtypes of breast cancer. These findings support the concept that in order to understand CCL2 expression as tumor promoting or suppressive factor, one must use additional molecular or cellular features to identify either the cells, or intrinsic subtype of the breast cancer expressing CCL2 to determine whether CCL2 is playing a significant role in RFS.

While we expected that the delivery of exogenous CCL2 might reduce the outgrowth of the tumor cells based upon Granot’s work, we observed the opposite, potentially because there was no significant increase in neutrophil recruitment the lung. When we delivered 100 ng exogenous CCL2 daily by the intranasal route to mice, we did not observe a significant increase in the level of CCL2 at the end point of the experiment when the lungs were removed 2 weeks later. This is likely due to rapid clearance of the chemokine through the lung and to the uptake of CCL2 by the leukocytes, endothelial cells, and other cells in lung [28]. Though only subtle changes were observed in the infiltrating leukocyte populations in the lung in response to intranasal delivery of CCL2 (increased CD4+ T cells, increased central memory CD8+ T cells, increased CD206+ macrophages, and increased MHCII expressing macrophages), the end result was enhanced outgrowth of the 67NR cells in the lung. Our data are in accordance with the work from the Pollard laboratory and other groups showing that CCL2 can promote metastasis [6, 9, 39–43]. There are reports that the tumor promoting effect of CCL2 applies to both ER+ and triple negative breast cancers [39, 44]. Taken together, our data suggest that while less aggressive tumors may indeed alter the microenvironment of the pre-metastatic niche of the lung to inhibit outgrowth of metastatic tumor cells, our attempts to mimic this with delivery of exogenous CCL2 resulted in enhanced seeding and outgrowth of breast cancer cells in the lung. Moreover, increased CCL2 produced by stromal cells in the tumor microenvironment has been reported to promote metastasis of 4T1 TN breast cancer cells and ER+ breast cancer to the lung [35, 44] and to support a cancer stem cell phenotype [45]. In addition, CCL2-mediated activation of SMAD3 and the MAPK pathway are involved in the increased survival of tumor cells and an enhanced metastatic phenotype of these cells [46].

## Conclusions

In conclusion, the link between CCL2 in breast cancer metastasis remains obtuse. While there are some potential anti-tumor effects of CCL2 in vitro and in vivo during early tumor formation, there are strong data from many groups, including the data reported here, indicating that intra-tumoral CCL2 can promote breast tumor growth and/or metastases. Clinical trials are currently ongoing for solid tumor patients using CCL2/CCR2 inhibitors in combination with other therapies [13]. However, there are some concerns about this since in mouse lung cancer models, stopping anti-CCL2 therapy results in a rapid regrowth of tumor with enhanced metastasis [41]. The data reported here may be important in evaluating response to CCL2 or CCR2 inhibitors in these ongoing trials. While our data support a potential anti-tumor role for CCL2 in TEN-mediated tumor killing in the poorly aggressive 67NR BALB/c mouse tumor model, when CCL2 was delivered via the intranasal route, an increase in CCL2 associated CD4+ T cell and CD206+ macrophage recruitment was associated with enhanced seeding and growth of tumor cells in the lung. Thus evaluation of effects of CCL2/CCR2 inhibitor treatment in clinical trials on the specific subsets of intra-tumoral leukocytes may be informative for evaluating response to therapy.

## Additional file

Additional file 1: Figure S1. Naïve BALB/c neutrophils can kill PyMT (FVB) tumor cells, but CCL2 does not increase killing. PyMT cells from FVB mice seeded with and without naïve BALB/c neutrophils (30 neutrophils: 1 tumor cell), in the absence and presence of CCL2. After 18-h at 37 °C, cells were lysed and luciferase was measured to determine tumor cell killing. Although from a different mouse strain, naïve BALB/c neutrophils were able to kill FVB PyMT tumor cells (*p* = 0.005). However, CCL2 did not enhance this effect (*p* = 0.347), Kruskal-Wallis test with Dunn’s test for multiple comparisons. Values are graphed as mean ± SD. **Figure S2.** Entrainment properties of less aggressive PyMT tumor cells on the metastatic outgrowth of more aggressive TGFβR2 knock out PyMT tumors. Female FVB mice (10 weeks old) were injected with either 15,000 PyMT breast cancer cells (MFP) or PBS (Non-tumor bearing) in the 4^th^ mammary fat pad. Two weeks later either 1 × 10^6^ TGFβR2 knockout PyMT (TbR2KO) breast cancer cells or PBS alone (in 200 μl) were delivered by tail vein injection to mice bearing PyMT tumors or into non-tumor bearing mice (t.v. TbR2KO). Three weeks later, mice were sacrificed and lungs were removed, fixed, H&E stained and the number of metastases counted. Analysis of variance with blocking (two experiments) was performed for an overall comparison (*p* < 0.001). Tukey’s honestly significant difference (HSD) for multiple comparisons among groups (adj. *p* = 0.009 for MFP-PBS vs. MFP + TbR2KO, adj. *p* < 0.001 for MFP-PBS vs. t.v. TbR2KO). NS = not significant, *p* < 0.1, **p* < 0.05, ***p* < 0.01, ****p* < 0.001. Values are graphed as mean ± SD. **Figure S3.** Intranasal delivery of CCL2 facilitates the recruitment of leukocytes into BAL fluid. 3A. BAL fluid isolated from mice receiving intranasal delivery of CCL2 showed an increase in CD8+ T cells as CCL2 delivery increased from 100 ng to 1000 ng. Data are shown as % CD45+ cells and as % total cells. 3B. BAL fluid from mice receiving intranasal delivery of CCL2 exhibited a trend toward increased numbers of neutrophils and NK cells with increasing concentrations. Data are shown as % live cells in BAL fluid. **Figure S4.** Blocking antibody to CCL2 reverses the neutrophil killing of 67NR cells in vitro. 67NR cells(T) expressing luciferase were seeded with and without neutrophils (E for effector cells) at a ratio of 30 neutrophils: 1 tumor cell in the presence of control IgG or blocking antibody to CCL2. After an 18-hs at 37 °C, cells were lysed and luciferase was measured to determine tumor cell killing. Anti-CCL2 (50 ng/ml BD Biosciences) reversed the 67NR tumor cell killing of BALB/c TEN, *p* < 0.01, Student’s *t* test, *n* = 5 per group. (PPTX 124 kb)

## Abbreviations

CCL2: CC chemokine 2
CCL4: CC chemokine 4
CCR2: CC Chemokine Receptor 2
CXCL8: CXC chemokine 8
ELISA: Enzyme-linked immunosorbent assay
ER: Estrogen receptor
FBS: Fetal bovine serum
GFP2: Green fluorescent protein 2
H&E: Hematoxylin and Eosin
HSD: Honestly significant difference
IL-4: Interleukin 4
KO: Knock out
MCP-1: Macrophage chemotactic protein-1
MFP: Mammary fat pad
MHC: Major histocompatibility complex
MMTV: Mouse mammary tumor virus
NADPH: Nicotinamide adenine dinucleotide phosphate (reduced form)
Opti-MEM: Optimized minimal essential medium
PyMT: Polyoma Middle T antigen
RFS: Relapse-free survival
ROS: Reactive oxygen species
TbR2KO: TGF-β receptor 2 knock-out
TEN: Tumor entrained neutrophil
TGF-β: Transforming growth factor-beta
TGFβR2: Transforming growth factor-beta receptor 2
