# Supplementary material for: The Yin/Yan of CCL2: a minor role in neutrophil anti-tumor activity in vitro but a major role on the outgrowth of metastatic breast cancer lesions in the lung in vivo
Source: BMC Cancer. 2017 Jan 31;17:88. doi: 10.1186/s12885-017-3074-2 (PMC5286656; doi:10.1186/s12885-017-3074-2)
Supplement: Additional file 1: Figure S1. — Naïve BALB/c neutrophils can kill PyMT (FVB) tumor cells, but CCL2 does not increase killing. PyMT cells from FVB mice seeded with and without naïve BALB/c neutrophils (30 neutrophils: 1 tumor cell), in the absence and presence of CCL2. After 18-h at 37 °C, cells were lysed and luciferase was measured to determine tumor cell killing. Although from a different mouse strain, naïve BALB/c neutrophils were able to kill FVB PyMT tumor cells (p = 0.005). However, CCL2 did not enhance this effect (p = 0.347), Kruskal-Wallis test with Dunn’s test for multiple comparisons. Values are graphed as mean ± SD. Figure S2. Entrainment properties of less aggressive PyMT tumor cells on the metastatic outgrowth of more aggressive TGFβR2 knock out PyMT tumors. Female FVB mice (10 weeks old) were injected with either 15,000 PyMT breast cancer cells (MFP) or PBS (Non-tumor bearing) in the 4th mammary fat pad. Two weeks later either 1 × 10^6^ TGFβR2 knockout PyMT (TbR2KO) breast cancer cells or PBS alone (in 200 μl) were delivered by tail vein injection to mice bearing PyMT tumors or into non-tumor bearing mice (t.v. TbR2KO). Three weeks later, mice were sacrificed and lungs were removed, fixed, H&E stained and the number of metastases counted. Analysis of variance with blocking (two experiments) was performed for an overall comparison (p < 0.001). Tukey’s honestly significant difference (HSD) for multiple comparisons among groups (adj. p = 0.009 for MFP-PBS vs. MFP + TbR2KO, adj. p < 0.001 for MFP-PBS vs. t.v. TbR2KO). NS = not significant, p < 0.1, *p < 0.05, **p < 0.01, ***p < 0.001. Values are graphed as mean ± SD. Figure S3. Intranasal delivery of CCL2 facilitates the recruitment of leukocytes into BAL fluid. 3A. BAL fluid isolated from mice receiving intranasal delivery of CCL2 showed an increase in CD8+ T cells as CCL2 delivery increased from 100 ng to 1000 ng. Data are shown as % CD45+ cells and as % total cells. 3B. BAL fluid from mice receiving intranasal delivery of CCL2 exhibite [file 12885_2017_3074_MOESM1_ESM.pptx]

## Slide 1
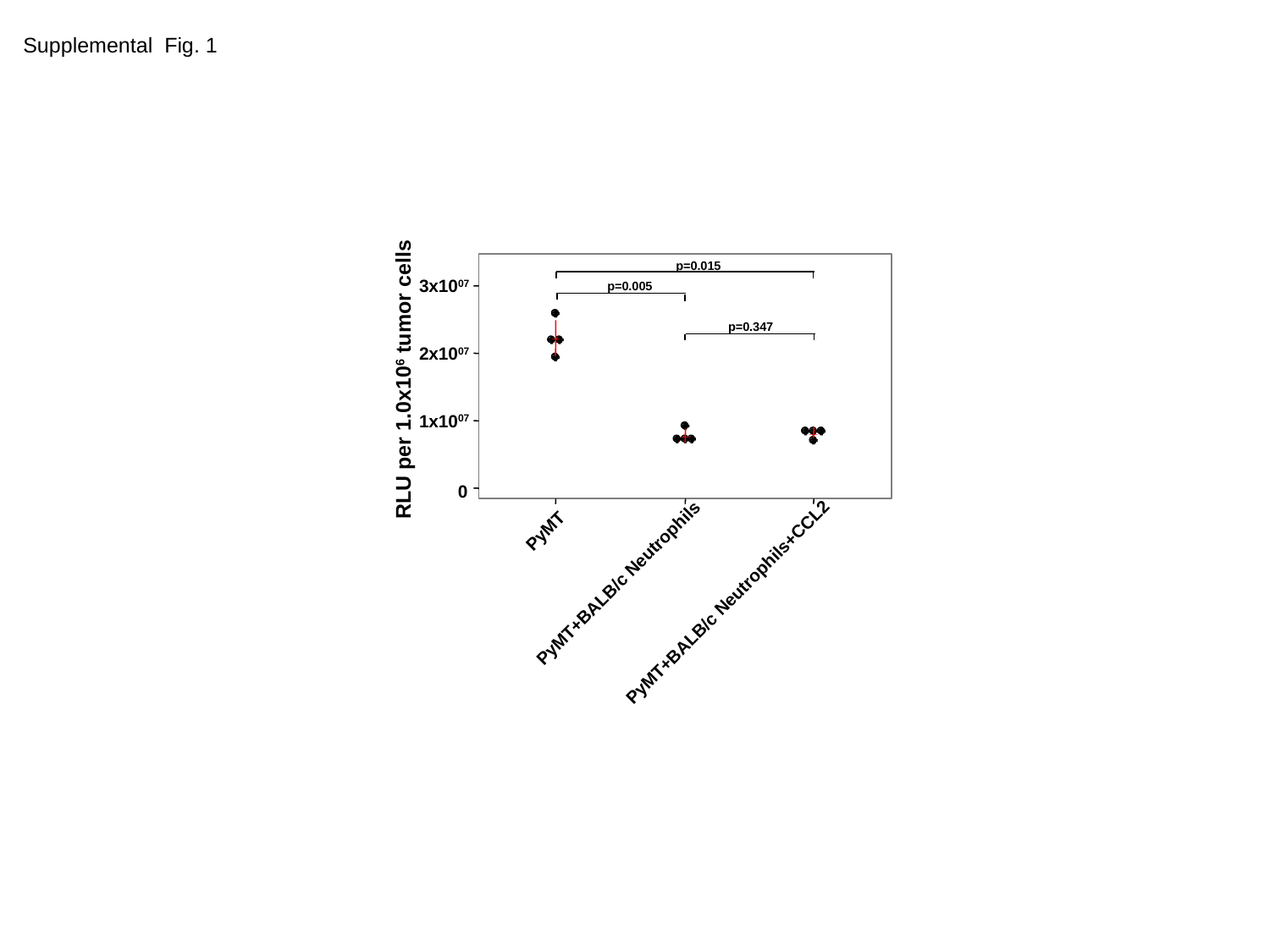

Supplemental Fig. 1
3x1007
_
2x1007
RLU per 1.0x10^6^ tumor cells
1x1007
_
_
 0
PyMT
PyMT+BALB/c Neutrophils
PyMT+BALB/c Neutrophils+CCL2
p=0.015
p=0.005
p=0.347

## Slide 2
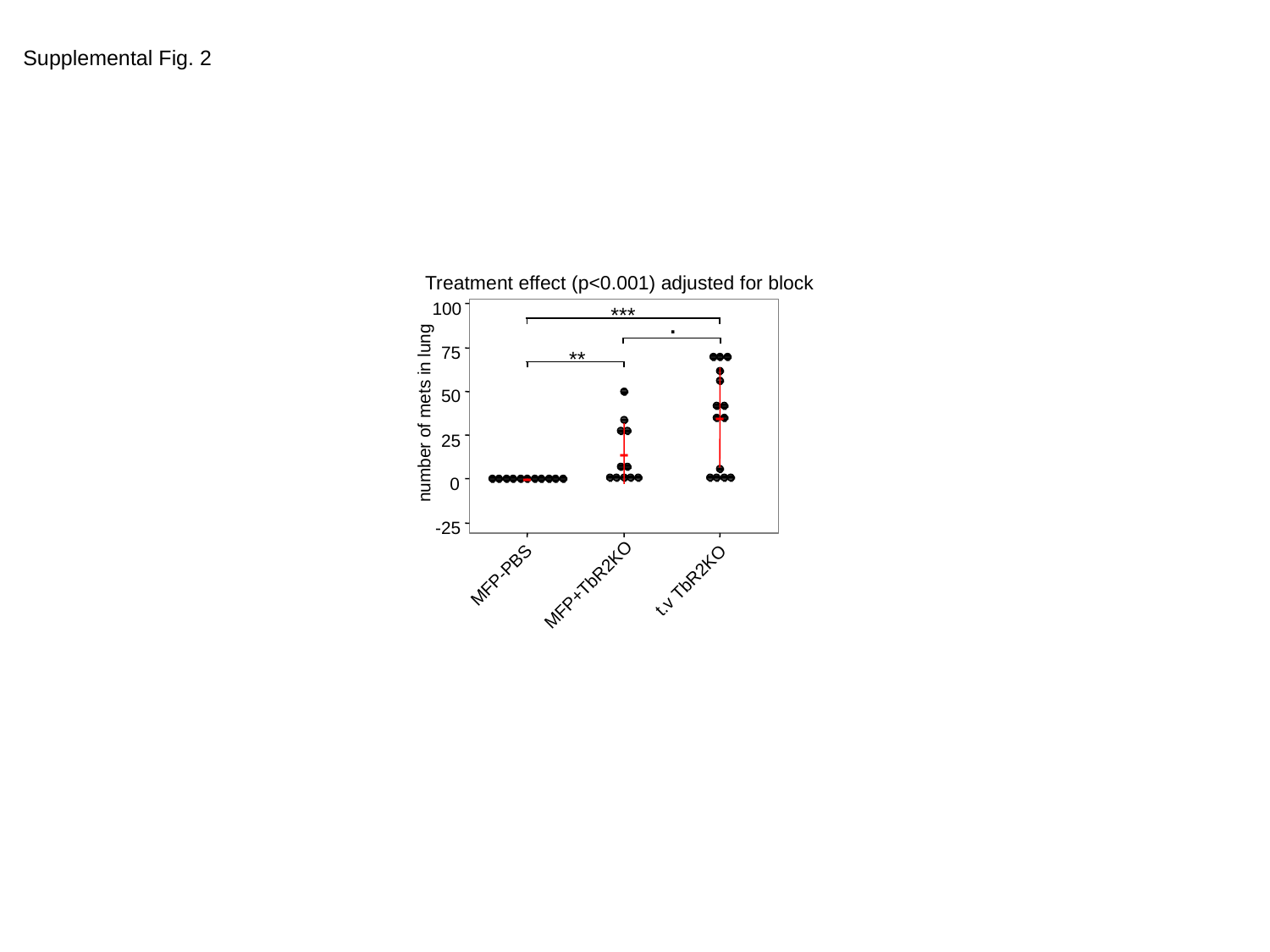

Supplemental Fig. 2
Treatment effect (p<0.001) adjusted for block
100
75
50
-
number of mets in lung
25
-
-
0
-25
MFP-PBS
t.v TbR2KO
MFP+TbR2KO
***
.
**

## Slide 3
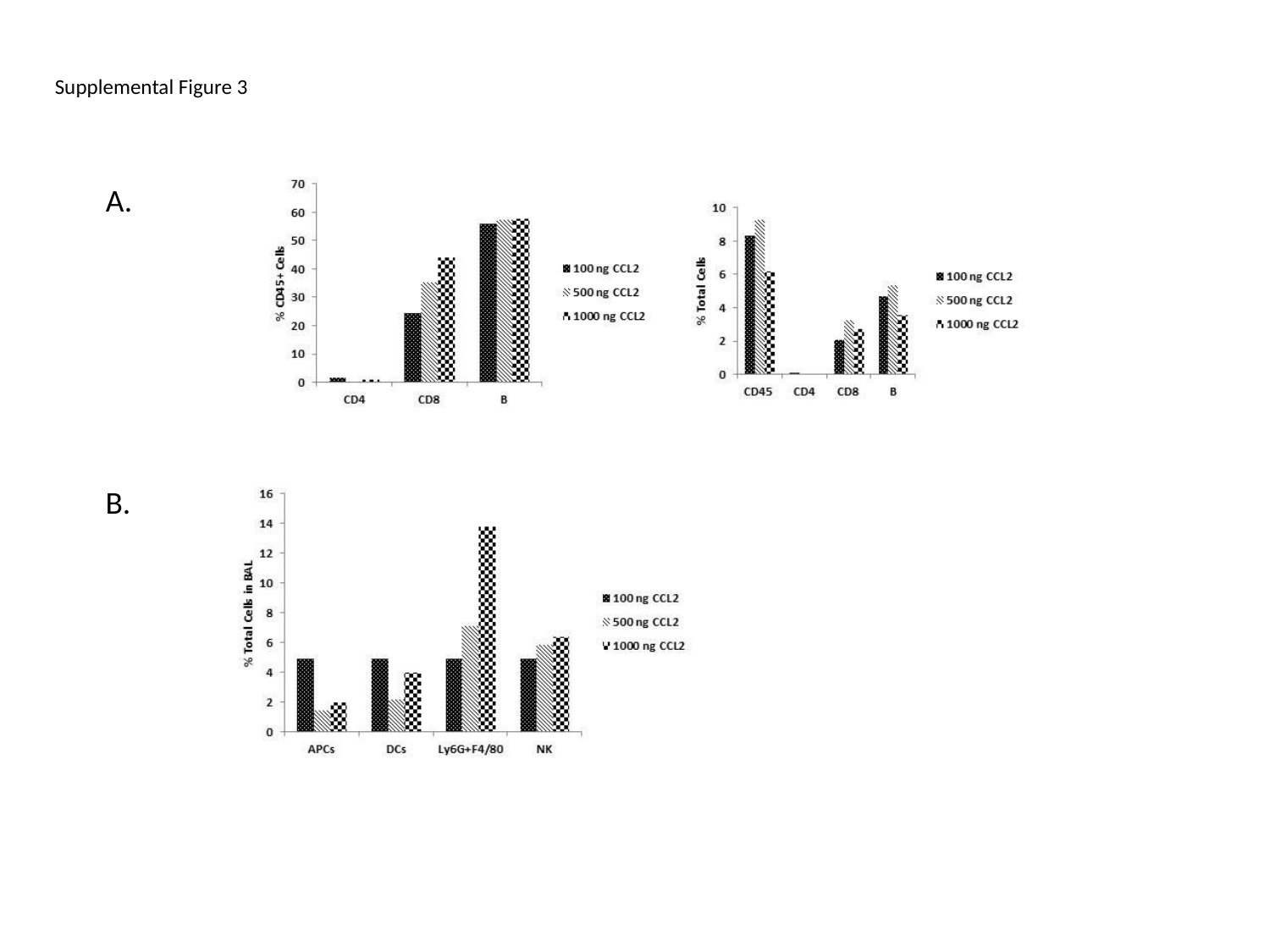

Supplemental Figure 3
A.
B.

## Slide 4
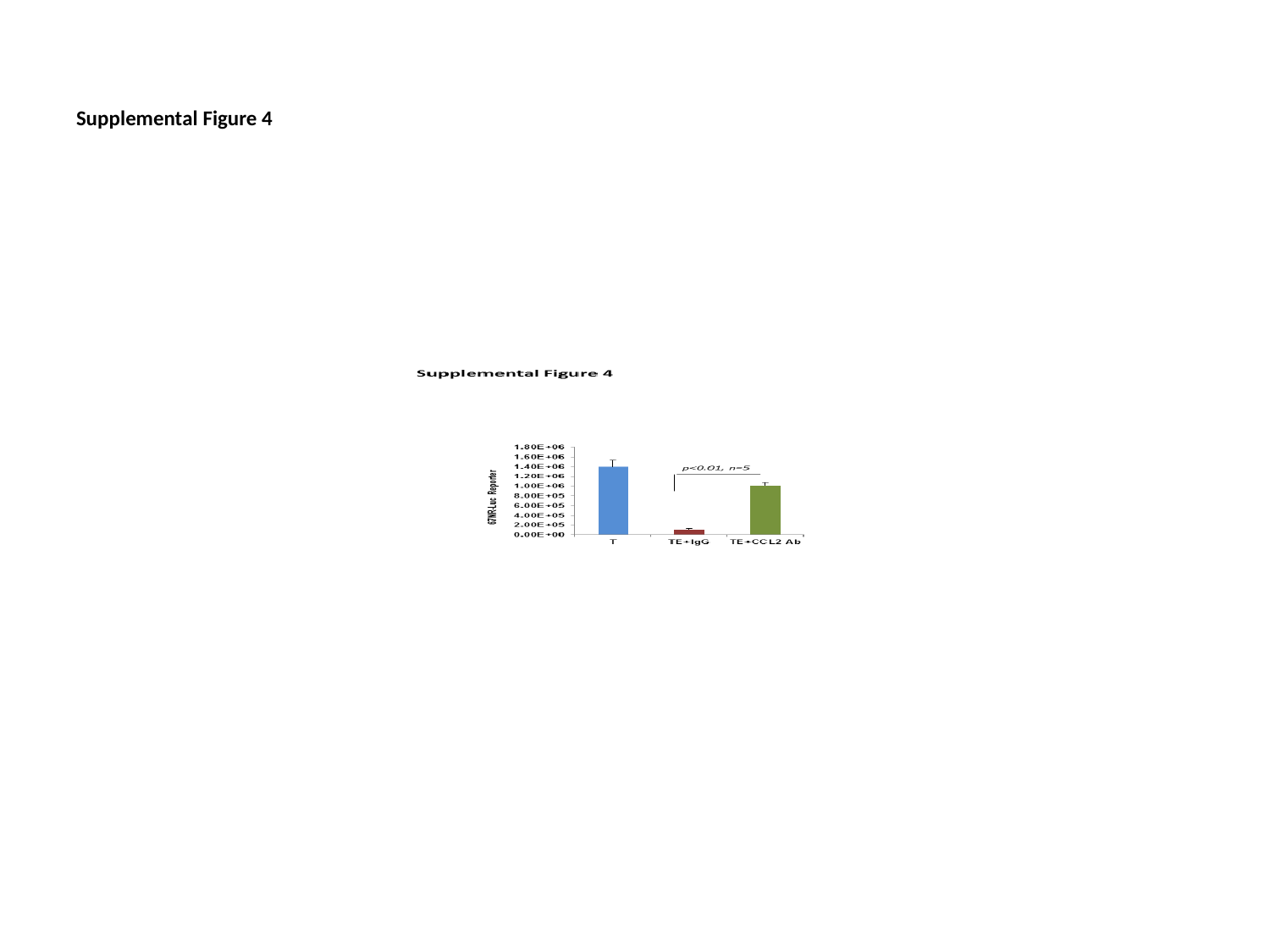

# Supplemental Figure 4
